# Targeting the Milieu in Neurodegenerative Disease: Time for “Imprecision Medicine”?

**DOI:** 10.14336/AD.2025.0966

**Published:** 2026-09-11

**Authors:** Joseph F Quinn

**Affiliations:** ^1^Department of Neurology, Oregon Health and Science University, Portland VA Medical Center PADRECC, Portland, OR 97239, USA.

**Keywords:** Alzheimer's, Parkinson's, precision medicine

## Abstract

The possibility of developing precision therapies for neurodegenerative disease is tantalizing, and efforts are under way with several diseases, including Alzheimer’s, Parkinson’s and Huntington’s disease. Despite strong basic neuroscience foundations and excellent clinical trial design, however, these efforts have been disappointing. We present an argument for a complementary approach of targeting the brain milieu in order to achieve disease-modifying effects in patients with or at risk of neurodegenerative disease. We suggest that a milieu-directed “brain health” approach can be applied across a range of at-risk individuals in a specific, quantitative, evidence-based manner. In support of this position, we present data from epidemiologic studies and clinical trials. We propose a program of rigorous research to validate and implement this complement to precision medicine which skeptics might call “imprecision medicine.”

## The central dogma of neurodegeneration and the promise of precision medicine

**Figure 1.**
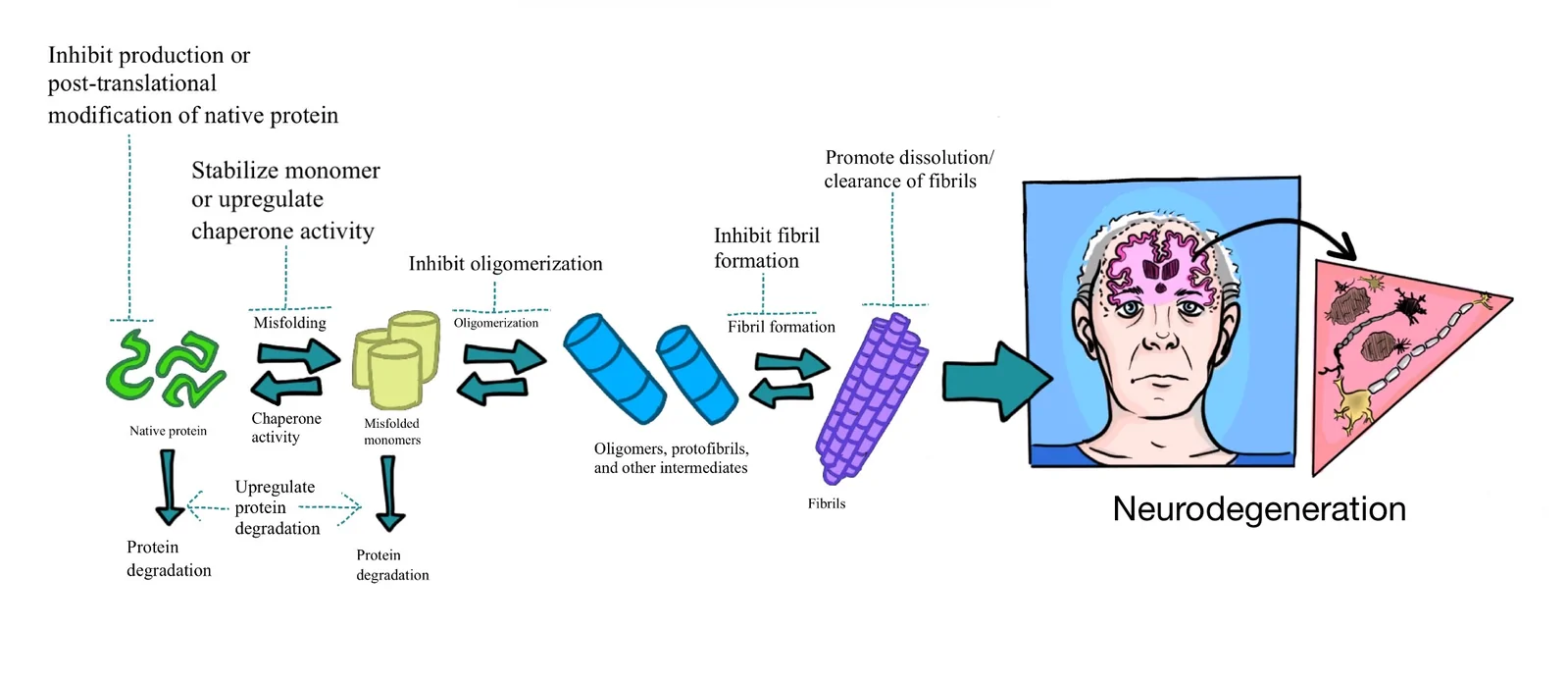
**The Central Pathway/Dogma of Neurodegeneration**. The “central pathway” illustrates the “central dogma” of neurodegeneration, in which protein misfolding events, governed by expression of the parent protein, post-translational modification (e.g., methylation, acetylation), biophysical events governing aggregation, and clearance rates, ultimately lead to neuronal damage and neurodegeneration. This general schema is cited for Alzheimer’s, Parkinson’s, Huntington’s, ALS, and other neurogenerative diseases. Precision medicine approaches to date have focused on these “central pathway” events with only limited success.

“Precision medicine,” meaning the tailoring of therapy to an individual’s genetics or other individualized characteristics, has seen great success in oncology. There is great potential and great enthusiasm for a similar approach to neurodegenerative disease. However, efforts to date in clinical neuroscience have been mostly disappointing. We recognize that these initial efforts have yielded important lessons, and we are optimistic that refinements of the approach will eventually yield success in neuroscience comparable to what has been seen in oncology. At the same time, however, we propose here a complementary approach focused not on the central pathways of neurodegeneration but on the surrounding milieu of the brain. By “central pathways”, we mean the widely accepted model of neurodegeneration in which protein misfolding leads, in a linear fashion, to neuronal injury and neurodegeneration (Fig. 1)

Disappointing negative results with “precision” approaches have now been seen with Parkinson’s disease, Huntington’s disease, and Alzheimer’s disease. In the case of Parkinson’s disease, two Phase 2 studies of antibodies directed against aggregated alpha synuclein have failed [1, 2]. A clinical trial of a therapy targeting the glucocerebrosidase (GBA) pathway in PD patients carrying the GBA genetic risk factor has failed [3].

In Huntington’s disease, the failed trials are especially disappointing, as the HD gene provides great diagnostic certainty, and the intervention to suppress the mutant gene expression with anti-sense oligonucleotides is a particularly powerful and precise approach—and yet the trials failed [4].

In Alzheimer’s disease, precision approaches have included inhibitors of the enzymes responsible for generating beta amyloid from the precursor protein as well as many trials of anti-amyloid antibody therapy. The enzyme inhibitor trials each showed a trend to worse outcomes in the treated patients [5-7].

The anti-amyloid antibody trials illustrate both the utility and the limitations of the precision medicine approach. For example, the early studies of bapineuzamab [8] pre-dated the widespread availability of amyloid PET scans, so the study population was likely contaminated by individuals without amyloid pathology. The advent of amyloid PET scans was particularly helpful for confirming the diagnosis in individuals at the prodromal mild cognitive impairment phase of disease, which permitted subsequent demonstration that antibody therapy is efficacious at these early phases. The amyloid PET scan also facilitated precision in dosing, as serial PET scans permit confirmation of target engagement in a manner that was not possible previously.

Nevertheless, it required thousands of participants in each trial to demonstrate the modest clinical efficacy of aducanumab [9], lecanemab [10], and donanemab [11], which many experts and patients consider too small to be worth the risks, the cost, and the burden of therapy [12, 13]. In the case of each antibody, the effect on cerebral Aβ is profound, but the effect on clinically important outcome measures is quite modest. These studies argue that the mechanisms of neurodegeneration are not limited to the downstream effects of pathological Ab. Other self-perpetuating events involving pathological tau have been invoked to explain the limited clinical efficacy of anti-amyloid therapy, but we hypothesize that additional milieu factors are at play in this and other failed precision therapy efforts described above.

This hypothesis is also consistent with observations that genetic variants distinct from the “central pathway” have important modifying effects on neurodegeneration. One example is the robust effect of the Christchurch apolipoprotein E variant upon the expression of autosomal dominant Alzheimer’s disease [14]. Another example is seen in the apparent “protective” effect of the Parkinson’s-associated LRRK2 mutation upon neurodegeneration in individuals with the Parkinson’s-associated GBA mutation [15].

## Targeting the mileu rather than the central pathway

**Figure 2.**
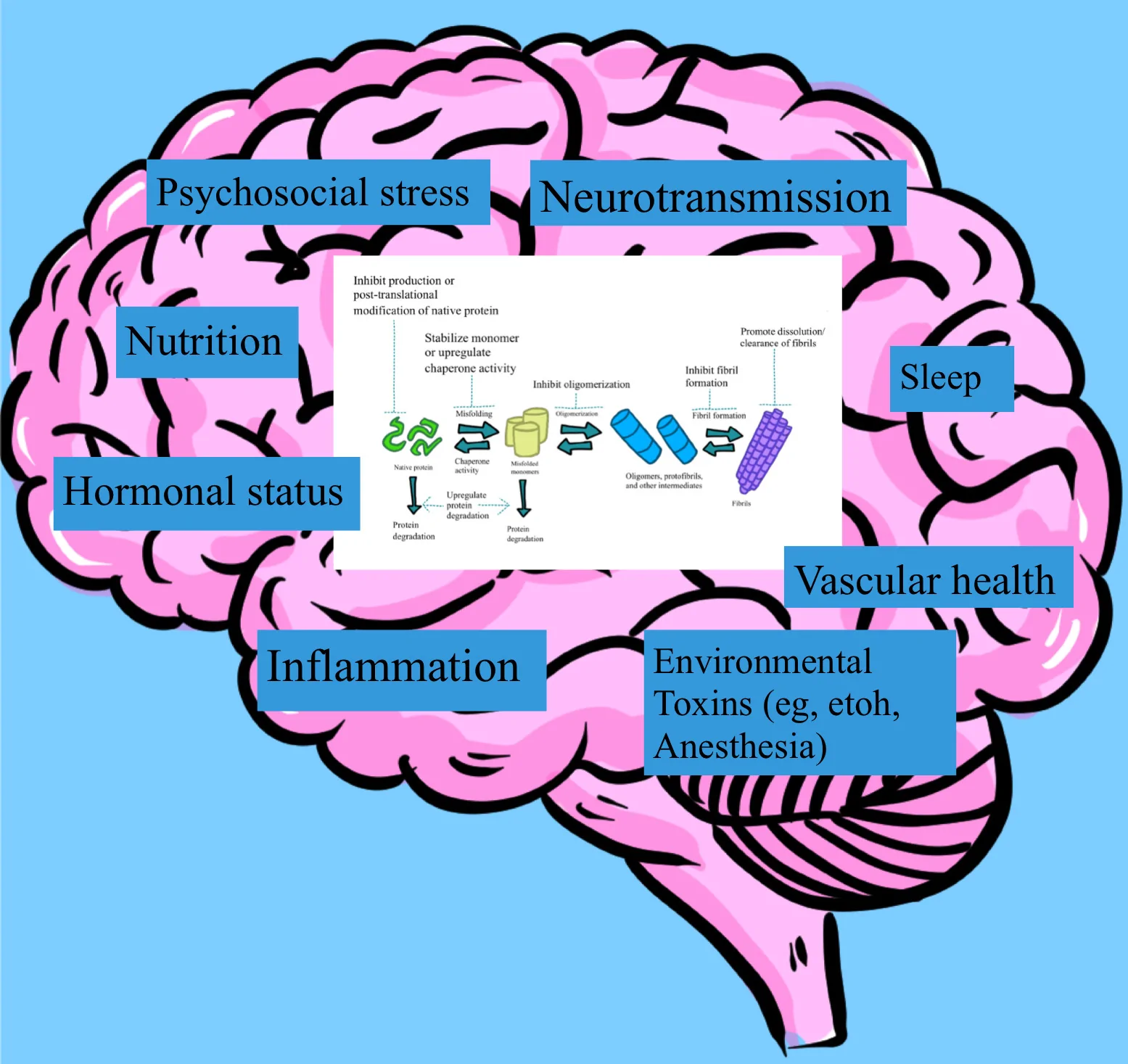
**Milieu factors modifying expression of the Central Pathway**. This figure emphasizes that the “central pathway” of neurodegeneration operates in a brain environment comprising discrete “milieu factors” (vascular, hormonal, stress-related, sleep-related, etc) which modify the ultimate outcome of protein misfolding events. The figure separates the “central pathway” from the milieu factors in order to emphasize that these factors do not necessarily modify protein mis-folding, aggregation, or the primary mechanisms of neurotoxicity but instead modify the milieu to allow the whole brain and body to cope with the neurodegenerative cascade in a manner that preserves synapses, neurons, and function. We propose here a program of research deliberately targeting these milieu factors to improve neurologic outcomes.

We hypothesize that the brain milieu modifies the expression of neurodegenerative disease in a clinically important way, since the “central pathway” described above plays out in the milieu of vascular health, nutritional health, inflammation, the endocrine system, and other brain milieu factors as illustrated in Figure 2.

Epidemiologic literature supports the concept that brain milieu factors are important for the expression of neurodegenerative disease. For example, we know from countless epidemiologic studies that vascular risk factors affect the risk of developing AD in a very significant way. For example, the latest report of the Lancet Commission on Dementia cites evidence that hypertension, smoking, obesity, physical inactivity, and diabetes are all risk factors for late life dementia including Alzheimer’s disease [16]. Several other studies have also documented that control of the vascular risk factors included in the American Heart Association’s “Life’s Simple 7” is associated with reduced risk of dementia [17-24]. While there may be important “downstream” effects of vascular dysfunction upon the generation of beta amyloid or tau pathology [25], an equally plausible explanation is that vascular dysfunction has an additive or synergistic effect in combination with Abeta. A memorable demonstration of this phenomenon comes from the “nun study”, which showed that participants with equivalent amounts of brain AD pathology varied in their expression of dementia [26]. Those harboring concomitant cerebrovascular disease had clinically manifest dementia, while those with no cerebrovascular disease had “silent” AD pathology [26]. Neuropathological series of late life dementia have shown that dual pathology (vascular and neurodegenerative pathology) is the rule rather than the exception in late life dementia [27], providing further evidence that cerebrovascular disease can “unmask” neurodegenerative disease. If vascular disease modifies disease expression, then vascular disease is an appropriate additional target for neuroprotection. Additional literature illustrates the effects of non-vascular milieu factors like sleep [28-30], psychosocial stress [31-33], and hormonal status [34, 35]upon the expression of dementia, illustrating the potential role for milieu factors that are not directly linked to the “central pathway” of protein misfolding.

The modifying role of cerebrovascular disease is also evident in the Parkinson’s disease literature linking white matter changes to late complications like dementia [36] and gait impairment [37, 38] in PD. Sleep [39], sex hormones [40], and other milieu factors [41] have also been implicated as modifiers of PD expression or outcomes. The most dramatic example may be the effect of physical exercise upon outcomes in PD. While exercise has both vascular [42, 43] and non-vascular [44, 45] effects on brain health, there is little evidence that exercise influences the “central pathway”, but instead is another clinically important “milieu factor.”

While the existence of multiple age-associated pathologies complicates matters in late life diseases like AD and PD [27], milieu factors are also important in younger onset diseases like Huntington’s disease. An abundance of evidence shows that environmental manipulations can alter pathology expression in transgenic HD mice [46, 47]. In human subjects, the influence of environmental factors has been implied by the incomplete explanation of age of onset by CAG repeat length [48]. Roles for modifying age of onset or severity of HD in human subjects have been demonstrated for substance abuse [49-52], sleep dysfunction [53], psychosocial stress hormone levels [54, 55], and frailty [56], supporting the hypothesis that milieu factors modify clinical outcomes even in genetically determined diseases.

## Milieu-directed therapies are effective

While the evidence above provides theoretical support for targeting the milieu in neurodegenerative disease, the real test may be how milieu-directed treatment strategies perform in placebo-controlled trials. There are excellent examples of this type of clinically important success in the cases of Friedreich’s ataxia, Amyotrophic lateral sclerosis, and Huntington’s disease.

Friedreich’s ataxia is an autosomal dominant spinocerebellar ataxia which is due to a mutation in the frataxin gene. The only FDA-approved treatment for Friedreich’s ataxia, omaveloxolone, is not directed at the gene or gene product, but instead targets the transcription factor Nuclear factor erythroid 2-related factor 2 (NRF-2) [57], which is not part of the central disease process of FA. NRF-2 instead modulates the oxidative milieu, by promoting the expression genes involved in antioxidant defense and mitochondrial biogenesis [58, 59]. In the absence of any demonstrable effect on frataxin or any other element of the “central pathway” of Friedreich’s neurodegeneration, omaveloxolone produced statistically significant benefits on standardized, clinically relevant scales like the modified Friedreich's Ataxia Rating Scale in double-blind placebo-controlled trials [60-62].

Amyotrophic lateral sclerosis (ALS) is associated in a minority of cases with known causative genes including superoxide dismutase 1 (SOD1) and C9ORF72. A placebo-controlled clinical trial targeting SOD1-associated ALS with a targeted anti-sense oligomer resulted in improvements in relevant biomarkers (cerebrospinal fluid SOD1 and plasma neurofilament light) but did not improve clinical endpoints [63]. A placebo-controlled trial targeting C9ORF72-associated ALS was also recently reported, and in that case neither clinical endpoints nor biomarkers were affected by treatment [64]. In contrast, edaravone, an agent which targets free radicals and oxidative stress in a non-specific manner, has shown clinically important benefits on survival and in function in placebo-controlled trials [65, 66] which were robust enough to justify FDA approval of this milieu-directed agent for ALS.

The final example to be described here is not yet FDA-approved but is in late-stage clinical development, with sufficient published data to cite it as another example of clinically productive targeting of the brain milieu rather than disease-specific molecular events. The disease in this case is Huntington’s disease, and the therapeutic target is not huntingtin, but instead the “sigma 1” receptor, which is not specifically involved in the neurodegenerative process. The drug pridopidine was originally investigated as a “dopamine stabilizer” in preclinical models of HD, but its beneficial effects were later shown to be mediated by the sigma 1 receptor [67], which is a chaperone protein localized in mitochondria-associated endoplasmic reticulum (ER) membranes, regulating Ca^2+^ signaling, reactive oxygen species (ROS) and mitochondrial fission [68]. In early controlled clinical trials in Huntington’s disease, pridopidine showed efficacy on motor outcomes and overall function [69-72], justifying Phase 3 trials. Although it did not reach its primary endpoint in Phase 3, an analysis of the participants who were not receiving anti-dopaminergic agents showed a statistically significant improvement from baseline in the composite unified Huntington’s disease rating scale, a combined measure of motor function, cognition, and functional capacity [73]. Since the long term safety profile is excellent [72], clinical development of pridopidine for Huntington’s disease continues, and other applications like ALS [74, 75], Alzheimer’s [76], and spinal cord ischemia-re-perfusion injury [77] are all under investigation.

## The implication of successful targeting brain milieu factors: A call for a complement to ongoing precision medicine trials

These examples illustrate the potential for improving outcomes in neurodegenerative disease by targeting brain milieu factors as a complement to the precision medicine efforts currently targeting the “central pathway” of neurodegeneration. This concept has implications for both pre-clinical and clinical efforts. For example:

1. Research targeting milieu factors in preclinical models is often dismissed because of a bias to focus on the central pathways of degeneration. Several milieu targets are worth citing as appropriate topics for rigorous research:
  - NRF2: This target has already been partially validated in the case of omaveloxolone for FA and dimethyl fumarate for multiple sclerosis, and NRF2 activators are under study for both neurologic [58, 78] and renal disease [79]. However, concerns about off-target effects of certain classes of NRF2 activators persist [80], and additional preclinical research will be necessary to optimally exploit this target in neurodegenerative disease.
  - Sigma 1 receptor: This target has also been partially validated in the case of pridopidine as noted above. Additional preclinical work has suggested a potential therapeutic role in Alzheimer’s, Parkinson’s, ALS, and stroke [81-83]. Translational work targeting this receptor is expected to be optimized by the development of PET imaging ligands for the receptor [84-86].
  - irisin is a hormone-like protein which is released from skeletal muscle during exercise, and is thought to mediate at least some of the neuroprotective effects of physical exercise, by crossing the blood-brain barrier and promoting the synthesis of brain-derived neurotrophic factor (BDNF) in the central nervous system [87]. The cognition-enhancing effects of irisin are well established, and research into the up-regulation of irisin to modify the course of Parkinson’s disease continues. However, an important potential adverse effect of irisin has been identified, namely the promotion of hepatocellular cancer [88]. Additional research into the regulation and the mechanism of action of irisin will be necessary in order to dissect the benefits from the risks of irisin in a manner that will permit clinical application.
  - alpha-klotho is a protein which serves as a biomarker of aging and which may mediate anti-aging effects, as klotho knockout accelerates aging while klotho upregulation prolongs lifespan [89]. Neuroprotectant effects of alpha-klotho have been reported in preclinical models, and clinical studies have shown correlations between circulating levels of alpha klotho and neurologic function [90] [91, 92]. Additional preclinical studies of alpha klotho may permit therapeutic application of this molecule for the prevention or treatment of age-associated neurodegenerative diseases like Alzheimer’s and Parkinson’s disease.
  - The Y-box binding protein (YBX-1) [93]is another protein with a variety of anti-aging properties. YBX-1 is important for learning and memory in c. elegans [94], and increased levels of this protein have beneficial effects in models of Alzheimer’s disease [95-97], but most of the research on this target is in oncology, and additional translational neuroscience research will be necessary to exploit the neurotherapeutic potential of this target.
  Promoting research into these “milieu factors” will require re-orientation of grant reviewers, possibly at the level of Requests for Applications (RFA’s) directed at this type of investigation. Otherwise, the practice of dismissing proposals that are not focused on the “central pathway” will continue, and opportunities for clinically meaningful intervention will be missed.
2. Clinical research, including clinical trials targeting milieu factors, should also be supported, regardless of evidence for disease-specificity. A priority should be the development of biomarkers of milieu target engagement, following the examples of blood measures of NRF2 activation [98], peripheral [99] and CNS biomarkers [100] of mitochondrial function, and PET imaging of sigma 1 receptor occupancy [101]. Another priority should be “exposome” research to identify environmental protective factors in a sort of reversal of the typical approach to environmental toxicology.
  Both clinical practice and clinical research should also focus on the promotion of healthy behaviors targeting validated milieu factors. Several of these (e.g., vascular risk factors, healthy sleep, depression treatment) do not need further validation but do require updated care delivery models and research into the promotion of healthy behaviors. The situation is complicated by diverse populations which vary in health literacy, adherence, and access to care. These confounding issues will require attention both in terms of care models and in research.
3. Precision medicine approaches also need to pay attention to the “noise” created by milieu factors in order to optimize the design of precision medicine trials. The current trend for assembling “trial-ready cohorts” [102] may benefit from efforts to optimize validated milieu factors in these cohorts, both in terms of improved retention while awaiting randomization and reduced “noise” after randomization to precision experimental therapeutics.
  We anticipate that skeptics may want to label this approach as “imprecision medicine” so we are beating them to the punch by offering this term even though we are actually recommending a precisely targeted, evidence-based approach. We believe there is much to be gained from targeting brain milieu factors and emphasize that this approach serves as a complement rather than an alternative to conventional drug development approaches.
